# A Functional Polymorphism in the Promoter Region of Interleukin-12B Increases the Risk of Colorectal Cancer

**DOI:** 10.1155/2020/2091781

**Published:** 2020-02-21

**Authors:** Yabin Liu, Binghui Li, Lili Wang, Dexian Kong

**Affiliations:** ^1^Department of General Surgery, Fourth Affiliated Hospital, Hebei Medical University, Shijiazhuang, Hebei 050011, China; ^2^Department of General Surgery, Shijiazhuang Traditional Chinese Medical Hospital, Shijiazhuang, Hebei 050051, China; ^3^Department of Endocrinology, Fourth Affiliated Hospital, Hebei Medical University, Shijiazhuang, Hebei 050011, China

## Abstract

**Objective:**

To investigate whether the polymorphisms of interleukin-12B (*IL-12B*) were associated with the risk of developing colorectal cancer (CRC). *Patients and Methods*. Genotypes of rs17860508 and rs3212227 were determined by polymerase chain reaction with a direct sequencing method in 329 CRC patients and 342 matched healthy control subjects. The expression of *IL-12B*) were associated with the risk of developing colorectal cancer (CRC).

**Results:**

Compared with TTAGAG/TTAGAG genotype of rs17860508, the GC/GC and TTAGAG/GC genotypes may significantly increase the risk of CRC (OR = 1.81, 95% CI = 1.18–2.78; OR = 1.46, 95% CI = 1.01–2.12, respectively). Furthermore, the mRNA levels of *IL-12B*) were associated with the risk of developing colorectal cancer (CRC). *P*=0.009) and TTAGAG/TTAGAG (*P*=0.009) and TTAGAG/TTAGAG (

**Conclusion:**

These data suggested that the rs17860508 GC/GC genotype might upregulate *IL-12B* expression at the transcriptional level and thus increase the risk of CRC.*IL-12B*) were associated with the risk of developing colorectal cancer (CRC).

## 1. Introduction

Colorectal cancer (CRC) is the third most common cancer and the fourth leading cause of cancer-related mortality in both genders worldwide [1]. In the past 20 years, the incidence of CRC has been rising rapidly in China. Despite great advances in diagnosis and treatment, the 5-year survival rate of CRC is still less than 65% [2]. Many studies have been performed on the pathogenesis of CRC; however, its etiology remains poorly understood. Growing evidence indicates that inflammation is a critical molecular event in carcinogenesis, and inflammatory mediators (e.g., cytokines) can influence the growth and invasion of CRC [3–5].

Interleukin-12 (IL-12) is a key inflammatory cytokine that could promote the differentiation of Th1 cells, stimulate the secretion of interferon-*γ*, and form an interconnection between innate and acquired immunity [6, 7]. Clinical research indicated that the levels of serum IL-12 were related to disease severity in gastric cancer patients [8]. Meanwhile, administration of IL-12 in mouse models can inhibit tumorigenesis and induce regression of established tumors [9–11]. These data suggest that IL-12 seems to be a feasible candidate for cancer immunotherapy. IL-12 is a heterodimeric cytokine composed of IL-12p35 and IL-12p40. It is important to note that IL-12p40 is not only a component of IL-12 but also has intrinsic biological activity. The widely appreciated function of IL-12p40 is to act as an antagonist of IL-12 by competing at the IL-12 receptor [12]. IL-12p40 is encoded by *IL-12B*, and several polymorphisms have been identified in the *IL-12B* gene. Among them, two polymorphisms, rs17860508 in promoter region and rs3212227 in 3′ untranslated region of *IL-12B*, have been reported to be related to altered IL-12p40 expression in vitro [13, 14]. Association of these two functional polymorphisms and risk of cancers, such as cervical cancer [15], lung cancer [16], breast cancer [17], and gastric cancer [18], have been conducted. However, little is known about the contribution of *IL-12B* polymorphisms to CRC development in the northern Chinese population.

In the present study, we explored the role of the two functional polymorphisms in the risk of developing CRC in a northern Chinese population. Additionally, we also examined the levels of *IL-12B* mRNA in CRC tissues and their adjacent normal tissues.

## 2. Material and Methods

### 2.1. Study Population

In this case-control study, a total of 671 blood samples were recruited consecutively from the Fourth Hospital, Hebei Medical University, between January 2014 and December 2017. This consisted of 329 CRC patients and 342 matched normal samples. Fifty fresh CRC tissue specimens and adjacent normal tissues (≥10 cm away from the edge of the tumor) from the same patient were collected in RNAlater solution for mRNA expression analysis. Diagnosis of CRC was confirmed by histological examination. Detailed clinical and pathological information of the patients was collected from their medical charts. Exclusion criteria included patients with any personal or family history of cancer, preoperative radiotherapy or adjuvant chemotherapy, and recurrent CRC. Controls were selected from healthy volunteers who participated in the general health check-up at the Fourth Hospital, Hebei Medical University, during the same time period of case recruitment. Exclusion criteria for controls were a personal or family history of cancer or inflammatory diseases in the intestine.

All participants in this study were people of Han ethnicity in northern China and provided written informed consent. This study was performed with the approval of the Ethics Committees of the Fourth Hospital of Hebei Medical University.

### 2.2. DNA Extraction and Genotyping

Genomic DNA was extracted no more than 1 week after sampling the blood using traditional proteinase K (Merck, Darmstadt, Germany) digestion followed by phenol-chloroform extraction and ethanol precipitation according to the method described by Miller et al. [19]. Genotypes of rs17860508 and rs3212227 were determined by polymerase chain reaction (PCR) with a direct sequencing method. The primers used for PCR amplification of rs17860508 were F: 5′-TGC CCC TCG GGA CTG ACT AT-3′ and R: 5′-GCA GAC CTT CCT CGC CCA TA -3'. The primer sequences for rs3212227 were F: 5′- TGG CAT TCT CTT CCA GGT TCT -3′ and R: 5′- GGC AAC TTG AGA GCT GGA AA -3'. PCR was performed in a 50 *μ*L volume containing 100 ng of DNA template, 5 *μ*L of 10 × PCR buffer, 5 *μ*L of 25 mmol/L MgCl_2_, 1 U of Taq DNA polymerase (TIANGEN Biotech, Beijing, China), 1 *μ*L of 10 mmol/L dNTPs, and 1 *μ*L of 200 nM forward and reverse primers. The PCR conditions were 3 min at 94°C followed by 35 cycles of 15 sec at 94°C, 15 sec at 55°C, and 30 sec at 72°C, and the final step was at 72°C for 3 min to allow for the elongation of all PCR fragments. Direct sequencing was performed on the PCR products using the ABI3730XL automated sequencer (Applied Biosystems, Foster City, CA) according to the standard protocols.

### 2.3. Quantitative Real-Time Reverse Transcriptase-PCR (RT-qPCR)

Total RNA was isolated from CRC tissues and their adjacent normal tissues using the TRIzol-chloroform extraction method (GENERAY BIOTECH CO., LTD, China). cDNA was synthesized using the First Strand cDNA Synthesis kit (Thermo Fisher Scientific, USA). RT-qPCR reactions were performed using QuantiNova™ SYBR® Green PCR Kit (Qiagen, Shanghai, China). The primer sequences for RT-qPCR amplification of *IL-12B* were as follows: 5′-CCC TGA CAT TCT GCG TTC A-3' (forward) and 5′-AGG TCT TGT CCG TGA AGA CTC TA-3' (reverse). *GAPDH* was used as an internal control, and the primer sequences were as follows: 5′-ACC ACA GTC CAT GCC ATC AC-3' (forward) and 5′-TCC ACC ACC CTG TTG CTG TA-3' (reverse). Relative amount of *IL-12B* mRNA was calculated with the 2^−ΔΔCt^ method after normalization to *GAPDH*, and all experiments were repeated three times.

### 2.4. Statistical Analysis

SPSS statistical software package (version 21.0; SPSS Inc., Chicago, IL, USA) was used for all of the statistical calculations. Hardy-Weinberg equilibrium (HWE) was performed to compare the observed genotype frequencies with the expected genotype frequencies using the chi-square test. Demographic and clinical data between cases and controls were compared by Student's *t*-test and by the chi-square test. Genotype and allele frequencies were analyzed using chi-squared test. The odds ratio (OR) and 95% confidence interval (CI) were tested using an unconditional logistic regression model to assess the effect of the two polymorphisms on CRC risk. Differences of *IL-12B* mRNA levels between CRC tissues and adjacent normal controls were compared using the Wilcoxon signed-rank test, and differences of *IL-12B* mRNA levels in different genotypes were compared using the Mann-Whitney *U* test with Bonferroni correction. *P* < 0.05 was considered as a significant difference.

## 3. Results

### 3.1. Demographic Characteristics of the Study Participants

Detailed information about the study participants is summarized in Table 1. There were no significant differences between cases and controls in age and gender distribution (*P*=0.381 and 0.301, respectively).

### 3.2. Association of the Two Polymorphisms with the Risk of CRC

The genotype and allele frequencies of rs17860508 and rs3212227 in cases and controls are listed in Table 2. Genotype distributions of rs17860508 and rs3212227 in the controls did not significantly deviate from those expected for Hardy-Weinberg equilibrium (*P*=0.923 and 0.847, respectively). There were significant differences in the genotype distributions and allele frequencies of rs17860508 polymorphism between cases and controls. Compared with the rs17860508 TTAGAG/TTAGAG genotype, the GC/GC and TTAGAG/GC genotypes may significantly increase the risk of developing CRC (OR = 1.81, 95% CI = 1.18–2.78; OR = 1.46, 95% CI = 1.01–2.12, respectively). The carriers with rs17860508 GC allele had a significantly increased risk of CRC compared to those with rs17860508 TTAGAG allele (OR = 1.35, 95% CI = 1.09–1.67). However, no significant difference was found in the genotype distributions and allele frequencies of rs3212227 between cases and controls.

Stratified analyses of the two polymorphisms and clinical characteristics of CRC patients are shown in Tables 3 and 4. No significant association was observed between rs17860508 and rs3212227 polymorphisms and the clinical characteristics of CRC patients.

### 3.3. Association of the Two Polymorphisms with the Levels of *IL-12B* mRNA

**Figure 1:**
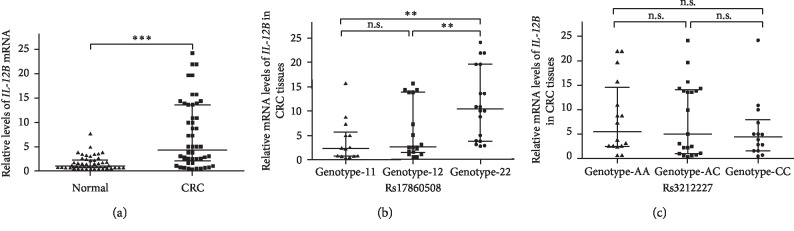
The mRNA expression of *IL-12B* in CRC and normal tissues. Data are expressed as median with interquartile range. (a) Relative mRNA levels of *IL-12B* in the 50 pairs of CRC tissues and their adjacent normal tissues. *∗∗∗P* < 0.001. (b) Relative mRNA levels of *IL-12B* in CRC tissues from patients carrying the different genotypes of rs17860508. TTAGAG allele was marked as 1, and GC was marked as 2. *∗∗P* < 0.01. (c) Relative mRNA levels of *IL-12B* in CRC tissues from patients carrying the different genotypes of rs3212227. CRC: colorectal cancer; n.s.: not significant.

The levels of *IL-12B* mRNA were measured using RT-qPCR in 50 pairs of CRC tissues and their adjacent normal tissues. The results showed that the levels of *IL-12B* mRNA were significantly increased in CRC tissues compared with adjacent normal tissues (*P* < 0.001, Figure 1(a)). Additionally, the levels of *IL-12B* mRNA were also analyzed in subjects with variant genotypes of *IL-12B* polymorphisms. As shown in Figure 1(b), the levels of *IL-12B* mRNA were significantly higher in the CRC tissues from patients with the rs17860508 GC/GC genotype than those with the TTAGAG/GC (*P*=0.009) and TTAGAG/TTAGAG (*P*=0.001) genotypes. However, there were no significant differences in the levels of *IL-12B* mRNA in CRC tissues from patients with the three genotypes of rs3212227 (Figure 1(c)).

## 4. Discussion

In this case-control study, we evaluated the association of genetic variants of *IL-12B* with the risk of developing CRC in a northern Chinese population. The results demonstrated that the rs17860508 polymorphism was associated with the risk of developing CRC. Compared with the TTAGAG/TTAGAG genotype of rs17860508, individuals with the GC/GC genotype had a higher risk of developing CRC. In addition, we found that the mRNA levels of IL-*12B* were significantly higher in CRC tissues than those in their paired adjacent normal tissues, and *IL-12B* mRNA levels were significantly different in CRC tissues from patients carrying different genotypes of rs17860508. However, the rs3212227 polymorphism may not be related to the risk of developing CRC.

The complex polymorphism of rs17860508 TTAGAG/GC is located in the promoter region of the *IL-12B* gene. Considerable evidence has revealed that this polymorphism could affect the expression of IL-12B. Shimokawa et al. [20] found that the transcriptional activity of the rs17860508 GC allele was higher than that of the TTAGAG allele in the human monocyte cell line. Hirota et al. [14] also demonstrated that the GC construct of rs17860508 could significantly enhance the transcriptional activity of *IL-12B* in a luciferase assay. The association between rs17860508 and the risk of human diseases has been examined in several studies, but the results were inconsistent. Naka et al. [21] showed that the rs17860508 TTAGAG allele was associated with significantly increased susceptibility to cerebral malaria in the Thai population. However, the frequency of the rs17860508 GC/GC genotype was significantly higher in the patients with systemic lupus erythematosus than that in the healthy subjects [22]. In this study, we found that the GC allele of rs17860508 was associated with higher susceptibility to CRC, and the GC/GC genotype carriers had higher levels of *IL-12B* mRNA in CRC tissues. Moreover, the levels of *IL-12B* mRNA in the CRC tissues were significantly higher than those in their paired adjacent normal tissues. Miteva et al. [23] also indicated that the IL-12p40 levels were significantly increased in sera from CRC patients compared to healthy donors. Although IL-12p40 is a monomer of bioactive heterodimer IL-12, it is worth mentioning that IL-12p40 in monomeric or homodimeric forms can act as a chemoattractant or as an IL-12 antagonist. IL-12 could stimulate the production of TNF-*α* and IFN-*γ*, which serves to mediate the destruction of cancerous cells by inducing an antiproliferative state. Therefore, we speculate that the rs17860508 GC/GC genotype may increase the expression of IL-12p40 by affecting the transcriptional activity of *IL-12B*, which may contribute to the risk of developing CRC.

Rs3212227 is an important functional polymorphism located in the 3ˊUTR of *IL-12B*. With regard to the genotypic and phenotypic functions of rs3212227, there were no consistent reports. Davoodi-Semiromi et al. [24] reported that the genotype of rs3212227 AA was associated with significantly elevated expression of IL-12 in peripheral lymphocytes from type 1 diabetes patients in Caucasian-American families. However, controversial results were observed by other studies [25, 26]. The association between rs3212227 polymorphism and the risk of cancers has been investigated in several studies, but the results were also inconsistent. The CC/AC genotypes of rs3212227 were associated with an increased risk of breast cancer [17], gastric cancer [18], bladder cancer [27], and cervical cancer [28]. However, there was no association between the rs3212227 polymorphism and the risk of hepatocellular carcinoma [29]. In the present study, we also failed to find a significant difference in the genotype distributions and allele frequencies of rs3212227 between CRC patients and controls. Furthermore, the levels of *IL-12B* mRNA were not significantly different in CRC tissues from patients with the three genotypes of rs3212227. These data suggested that rs3212227 may not play an important role in the susceptibility to CRC in our study population.

The main limitation of our study is the sample size, which comprised only 329 CRC patients and 342 control subjects. We could not eliminate the possibility that the positive results occurred by chance due to the relatively small sample size. Thus, larger population-based and multi-ethnic studies are needed to further confirm our findings.

## 5. Conclusion

Our data demonstrated that the rs17860508 polymorphism in the promoter of *IL-12B* was related to the risk of developing CRC in the northern Chinese population. The rs17860508 GC/GC genotype carriers had higher levels of *IL-12B* mRNA and increased susceptibility to CRC. These data suggest that the rs17860508 GC/GC genotype might upregulate *IL-12B* expression at the transcriptional level and thus increase the risk of CRC.

## Figures and Tables

**Table 1:** Clinical characteristics of study population.

Variable	Cases (*n* = 329)	Controls (*n* = 342)	*P*
Age, years (mean ± SD)	61.6 ± 10.2	60.8 ± 9.4	0.381
Gender			
Male	191 (58.1)	185 (54.1)	
Female	138 (41.9)	157 (45.9)	0.301
Differentiation			
Good + moderate	260 (79.0)		
Poor	69 (21.0)		
Clinical stage			
I-II	205 (62.3)		
III-IV	124 (37.7)		
Lymph node metastasis			
Yes	112 (34.0)		
No	217 (66.0)		
Tumor location			
Left-sided colon	227 (69.0)		
Right-sided colon	102 (31.0)		

**Table 2:** Association between the two polymorphisms and the risk of CRC.

Group	Controls (%)	Cases (%)	*P*	OR (95%CI)
	*N* = 342	*N* = 329		
rs17860508 genotype				
TTAGAG/TTAGAG	103 (30.1)	71 (21.6)		Reference
TTAGAG/GC	166 (48.5)	167 (50.8)	0.045	1.46 (1.01–2.12)
GC/GC	73 (21.3)	91 (27.6)	0.007	1.81 (1.18–2.78)
Allele				
TTAGAG	372 (54.4)	309 (47.0)		Reference
GC	312 (45.6)	349 (53.0)	0.007	1.35 (1.09–1.67)
rs3212227 genotype				
AA	108 (31.6)	96 (29.2)		Reference
AC	173 (50.6)	160 (48.6)	0.824	1.04 (0.73–1.48)
CC	61 (17.8)	73 (22.2)	0.182	1.35 (0.87–2.09)
Allele				
A	389 (56.9)	352 (53.5)		Reference
C	295 (43.1)	306 (46.5)	0.214	1.15 (0.92–1.42)

Abbreviations: CRC, colorectal cancer; OR, odds ratio; CI, confidence interval.

**Table 3:** Stratified analyses of the association between rs17860508 and clinical characteristics of CRC patients.

Clinical characteristics	Cases *n* = 329	TTAGAG/TTAGAG *n* (%)	TTAGAG/GC *n* (%)	GC/GC *n* (%)	*P*
Differentiation					
Good + moderate	260	60 (23.1)	129 (49.6)	71 (27.3)	
Poor	69	11 (15.9)	38 (55.1)	20 (29.0)	0.436
Clinical stage					
I-II	205	50 (24.4)	101 (49.3)	54 (26.3)	
III-IV	124	21 (16.9)	66 (53.2)	37 (29.8)	0.277
Lymph node metastasis					
Yes	112	20 (17.9)	58 (51.8)	34 (30.4)	
No	217	51 (23.5)	109 (50.2)	57 (26.3)	0.455
Tumor location					
Left-sided	227	45 (19.8)	114 (50.2)	68 (30.0)	
Right-sided	102	26 (25.5)	53 (52.0)	23 (22.5)	0.289

Abbreviations: CRC, colorectal cancer.

**Table 4:** Stratified analyses of the association between rs3212227 and clinical characteristics of CRC patients.

Clinical characteristics	Cases *n* = 329	AA *n* (%)	AC *n* (%)	CC *n* (%)	*P*
Differentiation					
Good + moderate	260	78 (30.0)	129 (49.6)	53 (20.4)	
Poor	69	18 (26.1)	31 (44.9)	20 (29.0)	0.309
Clinical stage					
I-II	205	62 (30.2)	104 (50.7)	39 (19.0)	
III-IV	124	34 (27.4)	56 (45.2)	34 (27.4)	0.206
Lymph node metastasis					
Yes	112	29 (25.9)	52 (46.4)	31 (27.7)	
No	217	67 (30.9)	108 (49.8)	42 (19.4)	0.212
Tumor location					
Left-sided	227	69 (30.4)	109 (48.0)	49 (21.6)	
Right-sided	102	27 (26.5)	51 (50.0)	24 (23.5)	0.760

Abbreviations: CRC, colorectal cancer.

## Data Availability

The data used to support the findings of this study are available from the corresponding author upon request.
